# Correspondence and Reply of Dr. W. Mitchell

**Published:** 1892-10

**Authors:** 


### Foreign Correspondence.

### CORRESPONDENCE AND REPLY OF DR. W. MITCHELL.

[The annexed correspondence will explain the reason why no answer has heretofore appeared to the criticism in *The Journal of the British Dental Association* on Dr. Mitchell's paper, published in the January number of this journal. The reasons given for the rejection of the reply are peculiar in view of the character and prolixity of the letters admitted to the pages of the journal named.—Ed.]

11 Queen Anne Street, Cavendish Square,
London, W., August 29, 1892.

W. Mitchell, Esq.:

Dear Sir,—At the request of the Publishing Committee of *The Journal of the British Dental Association* I am writing to say that they have fully considered your letter, and regret that under the present and constantly-recurring pressure of matter for the *Journal* they do not see their way to reopen a discussion upon the subject started by you, and which was practically closed by the replies inserted in the March number of the *Journal*, and they do not think that any good can accrue by again bringing the question forward.

They therefore regret their inability to insert your letter, which I beg to return.

Yours faithfully,
J. F. Colyer,
*Sub-Editor.*

39 Upper Brook Street, London, W.,
August 30, 1892.

J. F. Colyer, Esq.:

Dear Sir,—I must thank you for your kindly-written letter, for your Publishing Committee placed you in a very awkward position by their illogical action: 1st, by the delay in acting upon my paper as compared with the extreme haste with which the letters of my alleged critics were inserted; 2d, by refusing to give me barely one and one-fourth pages of *The Journal of the British Dental Association* when my would-be critics are allowed five pages; 3d, by refusing me the ordinary amenities of discussion in debarring me from closing that subject, an invariable rule at any organization where papers are read and subjects discussed pertaining thereto, thereby stamping as only traditional that much-vaunted virtue, "English fair play." You will please convey my regrets to the committee that their action was not based upon tangible grounds.

Hoping I have not intruded too much upon your valuable time, and again thanking you for your kind diplomacy in negotiating the onerous position your committee placed you in,

I remain, yours very truly,
W. Mitchell.

To the Editor of The Journal of the British Dental Association:

Sir,—A short time ago there appeared in your journal what purported to be criticisms upon a paragraph from a paper read by me at a dental meeting at Heidelberg. Had the paper been quoted *in extenso* instead of arbitrarily it would have furnished its own reply. But as the truth of my statements have been questioned, I must, in fair play, ask your indulgence in allowing me to verify them through your journal. With the personalities indulged in I have nothing to say, except I have yet to learn that they are an equivalent for argument, or that vituperation can take the place of logic. The anonymous side of the correspondence I cannot notice at all.

I am prepared to stand by and defend my paper as written and read before a European meeting, upon, in many respects, an essentially European subject, and can only conclude, by the vigorous manner in which it was assailed by your correspondents, as to its applicability. Apparently the greatest offence I committed in my paper was the question, "Why is it that the best and most progressive students, after completing their course on this side and having learned all their instructors were capable of imparting to them here, seek the halls of learning in America to secure that which they were unable to obtain here, where the best obtainable is supposed to be within easy reach?" As your correspondents have elected to so modify geography as to kindly consider England as constituting Europe, I can only add that, if the "best" do not go to the United States for advanced dental instruction, the most "progressive" *do.*

And for the benefit of those who would doubt the foregoing statement, the following data may furnish food for reflection.

From 1881 to 1891 there were in attendance at six representative dental colleges in the United States ninety-seven students from Great Britain, one hundred and seventy from British colonies, and three hundred and forty-two from continental Europe. In passing, would say, as five of your correspondents are connected with dental institutions, it would be interesting to know how many foreign students have entered the English dental schools during a like period.

In the face of the foregoing figures it will not do to say all these are deficient in capacity, and only represent the fringe of the profession, or that they went to the States because it was cheaper or more easy of access, or the requirements more superficial. The only logical deduction possible is that most of them went on popular judgment, or at the instigation of their preceptors, who, from definite knowledge, knew where that which was of the most importance to both dentist and public was obtainable,—viz., thorough practical knowledge.

That the foregoing is not an imaginative statement can be proven beyond the possibility of a doubt by reference to our literature as to the advancement made in conservative dentistry by the profession in America, the spirit of which is reflected in the teaching of the institutions of that country, to the advancement of our profession wherever dentistry is known. This is most substantially endorsed by ex-presidents of the Odontological Society and branches of the British Dental Association by sending their sons to the very institutions your correspondents would denounce, thus more effectually refuting than I could the bulk of the latter's arguments.

A reference by one of your correspondents having been made to the post-graduate courses held here a few years ago, a word or two in passing might not be out of place. These valuable adjuncts to dental practitioners have been a feature of American schools for the past fifteen years. Having attended a post-graduate course here, one thing that strongly impressed me was, that of the six lecturers at that school, three were graduates of American colleges, another had received his instruction at the hands of his father, who was an American dentist, and as no allusion whatever was made to the American system of dentistry, vol. ii. pages 831 and following, by one of the other lecturers whose text and illustrations were similar, I can only conclude in this case it is but another proof of the coincidence of action that occasionally occurs only in the case of truly great minds. I might add that at the same institution the chair of operative dentistry has been held for nearly, or quite, the past fifteen years by graduates of American colleges, and positions have been offered to at least two others. In another post-graduate course, held about the same time, we see in a leading journal that a demonstrator of the electric mallet "actually filled a crown cavity before the class." It has also come under my notice at dental meetings recently to hear metal crowns spoken of as "a passing fancy," and "a show of hands asked for from those who considered crown-work a legitimate operation." Such examples as these, coming as they do from high places, probably to quite an extent, furnishes a reply to my query at the opening of this communication.

I must, in the face of these facts, maintain that it is to the enterprise and capacity of American colleges, through their instructors and their noble ally, the press of that country, that the coming dentists must continue to look for the highest and most advanced ideals of conservative dental attainments.

Apologizing for intruding so much upon your valuable space,

I beg to remain, yours respectfully,

W. Mitchell.

Since writing the foregoing, a more potent argument, if possible, than any of the preceding has presented itself for the demolition of the statements of more than one of my supposed critics, and that is the winning of the Saunders prize by a man who had taken the degree of D.D.S., thereby proving the wisdom of his selection in taking the D.D.S. *first.* The Saunders scholarship is the highest prize at the disposal of the London Dental Hospital, and I see by Mr. Morton Smale's speech (see August number *Dental Record*, page 351), "some changes had been made in the method of deciding the award of the Saunders scholarship with a view to insure its being obtained by the best all-round man," thus clearly disposing of the statement that "the best students do not go to America," etc.

It may only be coincidental, yet for that reason may be worthy of mention, that the first report we have of crown-work being done at the London Dental Hospital was concurrent with the entrance of the aforesaid pupil to that institution (see *British Journal of Dental Science*, March 15, 1892, page 286).

